# Manual Versus Instrumental Rotation of the Fetal Head in Malposition at Birth (ROTATE Study): Protocol for a Randomized Controlled Trial

**DOI:** 10.2196/72505

**Published:** 2026-07-22

**Authors:** Dawn Parris, Raffaele Napolitano, Jonathan Bishop, Annette Briley, Laura Butler, Versha Cheed, Anna L David, Zaina Habib, Pollyanna Hardy, Kim Hinshaw, Laura L Jones, Rachel Lillywhite, Eleanor Molloy, Jacqueline Nicholls, Stephen O'Brien, Rachel Plachcinski, Andrew Shennan, Nigel Simpson, Maureen Treadwell, Bassel Wattar, Andrew Weeks, The ROTATE Study Group, Dimitrios Siassakos

**Affiliations:** 1 Maternal Fetal Medicine Elizabeth Garrett Anderson (EGA) Institute for Women's Health University College London London United Kingdom; 2 Birmingham Clinical Trials Unit University of Birmingham Birmingham United Kingdom; 3 Caring Futures Institute College of Nursing and Health Sciences Flinders University Adelaide Australia; 4 Department of Applied Health Sciences Birmingham Clinical Trials Unit University of Birmingham Birmingham United Kingdom; 5 EGA Institute for Women's Health University College London London United Kingdom; 6 Nuffield Department of Population Health National Perinatal Epidemiology Unit Clinical Trials Unit (NPEU CTU) University of Oxford Oxford United Kingdom; 7 Obstetrics and Gynaecology Sunderland Royal Hospital Sunderland United Kingdom; 8 Department of Applied Health Sciences School of Health Sciences University of Birmingham Birmingham United Kingdom; 9 Obstetrics and Gynaecology North Bristol NHS Trust Bristol United Kingdom; 10 National Childbirth Trust London United Kingdom; 11 Women and Children’s Health King's College London London United Kingdom; 12 School of Medicine University of Leeds Leeds United Kingdom; 13 Birth Trauma Association Derbyshire United Kingdom; 14 Clinical Trials Unit Anglia Ruskin University Cambridge United Kingdom; 15 Beginnings Assisted Conception Unit Epsom and St Helier University Hospitals NHS Trust London United Kingdom; 16 Comprehensive Clinical Trials Unit University College London London United Kingdom; 17 Women's and Children's Health Institute of Life Course & Medical Sciences University of Liverpool Liverpool United Kingdom

**Keywords:** rotational vaginal birth, Kielland forceps, vacuum extraction, manual rotation, obstetric forceps, obstetric anal sphincter injury, cesarean birth at full dilatation, randomized controlled trial, fetal malposition, qualitative process evaluation

## Abstract

**Background:**

Approximately 5% of women will be affected by fetal malposition at full cervical dilatation, with the occiput in transverse or posterior positions. These women are more likely to require assistance to give birth to their babies with either rotational vaginal birth or cesarean birth at full dilatation. Three different rotational methods can be used: rotational (Kielland) forceps, rotational vacuum, and manual rotation. Current evidence supporting the use of the 3 rotational methods is only from observational data. To date, no randomized controlled trial (RCT) of rotational methods has been completed.

**Objective:**

This study aimed to evaluate whether manual rotation of the fetal head in persistent malposition at full cervical dilatation reduces the risk of severe maternal perineal trauma without substantially increasing the risk of cesarean birth, compared with instrumental rotation.

**Methods:**

ROTATE is a pragmatic, multicenter, 2-arm parallel group, open-label RCT of manual versus instrumental rotation of the fetal head in malposition at birth with an internal pilot and an embedded qualitative process evaluation. The primary outcome is to evaluate whether manual versus instrumental rotation at full cervical dilatation reduces the risk of severe perineal trauma (superiority outcome), defined as a third- or fourth-degree tear, without substantially increasing the risk of cesarean birth at full dilatation (noninferiority coprimary outcome). A sample size calculation found that 4988 participants are required to detect a clinically meaningful reduction of third- or fourth-degree tears from 6% to 4% with 90% power (α=.05). A total sample size of 5200 participants from approximately 40 sites is anticipated, as loss to follow-up is expected to be about 4%. Neonatal trauma, a composite of potential outcomes relating to intrapartum hypoxia and physical trauma, is a safety signal. The setting is National Health Service consultant–led maternity units across the United Kingdom. Randomization is performed after eligibility has been confirmed and verbal consent has been obtained. Randomization is undertaken via a 24-hour telephone service or web-based system. Participants are randomized at an individual level on a 1:1 ratio to manual or instrumental (forceps or vacuum) rotation. Written consent is sought postnatally.

**Results:**

Data collection took place between September 9, 2022, and November 5, 2024. The total number of recruits is 321. The data analysis of primary and secondary outcomes is ongoing at the time of submission.

**Conclusions:**

A multicenter RCT of rotational methods has been conducted in the United Kingdom. Although the trial was closed early due to challenges in meeting recruitment targets, the results are still highly anticipated.

**Trial Registration:**

ISRCTN ISRCTN10193017; https://www.isrctn.com/ISRCTN10193017

**International Registered Report Identifier (IRRID):**

DERR1-10.2196/72505

## Introduction

### Background and Rationale

Within this document, we use the term *woman* for those giving birth for the purpose of brevity. However, it is important to acknowledge that not all those who give birth identify as women and that this work relates to all people giving birth, irrespective of gender identity.

Most vaginal births occur without complication or the requirement of medical intervention when babies are in an occipito-anterior position [1]. Fetal malposition at full cervical dilatation, defined as the fetal occiput in transverse or posterior positions, occurs in approximately 4% to 5% of all pregnant women [2]. There are 2 options for managing persistent fetal malposition in the second stage of labor: rotational vaginal birth or cesarean birth at full dilatation.

In total, 11% of women with fetal malposition in the first stage of labor will eventually undergo a rotational vaginal birth [3]. There are 3 different methods of rotational vaginal birth: rotational (Kielland) forceps, rotational vacuum, and manual rotation. Kielland forceps and rotational vacuum are both methods of instrumental rotation. Women are more likely to experience significant perineal trauma following rotational vaginal birth than cesarean birth at full dilatation, including obstetric anal sphincter injury (OASI; 10%) [2,4]. OASI can cause significant long-term problems, including urinary and anal incontinence, which can gravely impede quality of life [5]. Until now, evidence gleaned from observational studies has guided practice of rotational vaginal birth, with no multicenter randomized controlled trial (RCT) ever completed comparing outcomes, including the incidence of OASI, between different rotational methods. Compared to cesarean birth at full dilatation, rotational vaginal birth may be associated with risks for mothers and babies, including infection (1%-4%), vaginal wall lacerations (12%-20%), and neonatal trauma (2%-9%) [4].

Cesarean birth at full dilatation is the main alternative to rotational vaginal birth and does not expose the mother to the risk of OASI. However, compared to cesarean births performed before or during an earlier stage of labor, cesarean birth at full dilatation also carries greater risks to both mothers and babies. These risks include extended uterine incisions (20%); incision through the cervix (26%); postpartum hemorrhage (PPH; 10%-13%); hysterectomy (4%); prolonged hospital admission (49%); as well as impacted fetal head (33% of cesarean births at full dilatation, doubling in cases of failed attempts at assisted vaginal birth), neonatal skull fractures, and intracranial bleeds (1.3%) [6-11]. Women undergoing a cesarean birth at full dilatation are more likely to have a major hemorrhage and extended hospital stay than those following vaginal birth. Babies delivered by cesarean birth at full dilatation are more likely to require admission for intensive care than babies delivered by forceps [12]. Some risks of cesarean birth at full dilatation carry over into subsequent pregnancies with a greater risk of late miscarriage and preterm birth, which is recurrent and resistant to treatment [8]. It would be important to know whether any rotational method can reduce the risk of OASI in the mother, without increasing the rate of cesarean birth at full dilatation.

Estimates of the incidence rate of cesarean births at full dilatation at term range from 1.8% to 5% [13]. Globally, rates are rising, and this is thought to be due to both declining skills in assisted vaginal births and increasing hesitation in performing rotational vaginal births [14,15]. High-quality evidence from a multicenter RCT is urgently needed to evaluate the comparative safety and effectiveness of rotational methods. ROTATE is a once-in-a-lifetime trial of rotational methods, and such a study would be extremely difficult to replicate.

### Objectives

The primary objective of ROTATE is to evaluate whether manual rotation versus instrumental rotation of the fetal head in persistent malposition at full cervical dilatation decreases the risk of severe perineal trauma (OASI), without substantially increasing the risk of cesarean birth at full dilatation. The choice of instrument, Kielland rotational forceps or rotational vacuum, is decided by the operator based on their own experience and expertise or that of the obstetrician supervising them at the time of birth. Manual rotation may be followed by direct forceps, direct vacuum, or maternal effort.

Secondary objectives are as follows:

1. To evaluate whether there are differences between the 2 rotational methods in important additional clinical outcomes for women and babies, including severe neonatal trauma and morbidity as a safety signal (as described in the Outcome section); maternal outcomes include urinary and fecal incontinence and primary PPH
2. To compare the birth experience between the 2 different rotational methods using validated patient satisfaction and experience questionnaires
3. To establish a randomized cohort of women who have experienced fetal malposition at full cervical dilatation
4. To explore qualitatively the feasibility, acceptability, and appropriateness of the intervention and trial processes, including consent to participate in time-critical research, for women and health care professionals

The embedded qualitative process evaluation (QPE) will explore the feasibility, acceptability, and appropriateness of trial processes and interventions for women and health care professionals, to inform decision-making for progression from the internal pilot to a full trial. The QPE will be used iteratively to inform and finesse the trial process.

## Methods

### Trial Design

ROTATE is a pragmatic, multicenter, 2-arm parallel group, open-label RCT with an internal pilot and QPE. Participants are randomized to either manual or instrumental rotation at an individual level on a 1:1 ratio. The choice of rotational instrument, as discussed earlier, is at the discretion of the operator. In the United Kingdom, proficiency in different rotational methods varies, and few obstetricians report confidence in all 3 methods [16]. Therefore, randomization will be limited to 2 arms, as including all 3 methods would reduce the number of sites able to participate and the number of obstetricians willing to recruit. There is minimization by center and fetal position (occiput transverse or posterior) to ensure balance in treatment allocations. A total of 5200 participants will be randomized in a 3-year period from approximately 40 geographically diverse sites.

The internal pilot will last 9 months, and it will determine the feasibility of progression to the full trial. The decision to continue to a full trial will be based on predefined independent stop-go criteria (red, amber, and green traffic lights; Table 1). This will be supplemented by the QPE findings.

**Table 1:** Stop-go criteria (red, amber, and green) for progression from pilot to full trial.

Pilot (9 months)	Red	Amber	Green
Trial recruitment, %	<80	80-99	≥100
Recruitment rate, site, month	<5	5-6	>6
Number of sites opened (staggered)	<9	9-11	12
Total number of participants recruited	<350	350-432	≥437
Adherence of women to the randomized procedure, %	<90	90-95	>95
Follow up of women randomized—3 months, %	<80	80-95	>95
Written consent not received for women randomized, %	>10	5-10	<5

### Methods and Analysis

#### Study Setting

The study will be conducted at National Health Service consultant–led maternity units across the United Kingdom.

#### Eligibility Criteria

##### Main Trial

Eligibility criteria for the main trial are presented in Textbox 1.

**Eligibility criteria for the main trial.**

**Inclusion criteria**

- Women aged ≥16 years at the time of randomization
- A singleton pregnancy
- Women who have reached ≥36 weeks’ gestation
- Fetuses in a cephalic presentation
- Persistent malposition of the fetal head at full cervical dilatation, with the occiput between 2 o’clock and 10 o’clock (occiput transverse or posterior positions), which has been diagnosed either clinically or with intrapartum ultrasound, requiring rotational vaginal birth
- The rotational vaginal birth to be conducted or supervised by a single obstetrician or team who collectively have the following qualifications:
  Signed off as competent in both manual and instrumental rotation (Kielland rotational forceps and rotational vacuum) in accordance with the Royal College of Obstetricians and Gynecologists curriculum
  Good Clinical Practice trained

**Exclusion criteria**

- Women with contraindications to an assisted vaginal birth with either forceps or vacuum
- Women whose fetus’s occiput is between 11 o’clock and 1 o’clock (occiput anterior position)
- Fetuses in a brow presentation
- Intrauterine fetal death

##### Qualitative Process Evaluation

Women who have been approached about ROTATE, their birth partners, and health care professionals involved in the trial will be invited to take part in the QPE during the pilot phase (first 9 months from opening). The eligibility criteria are presented in Textbox 2.

At the time of the QPE, ROTATE did not have the provisions to provide translation services. An exclusion criterion for the RCT and therefore the QPE was women and their birth partners who are not able to respond to interview questions in English. Trial documents for the RCT are being translated into the 5 most spoken languages at ROTATE sites—Arabic, Polish, Romanian, Spanish, and Urdu. This means that the ability to communicate in English will no longer be an exclusion criterion to the RCT.

**Inclusion and exclusion criteria for ROTATE.**

**Inclusion criteria**

- Women
  Those who have been approached about ROTATE, whether they consent to the trial or not
  Eligible to take part in ROTATE
  Able to understand and respond to interview questions in English
- Birth partners
  Women who have consented for their birth partners to be contacted to be approached to take part in the qualitative process evaluation (QPE)
  Able to understand and respond to interview questions in English
- Health care professionals
  Clinicians involved in the recruitment and randomization of participants to ROTATE
  Midwives caring for women who have been approached about the trial

**Exclusion criteria**

- All
  Decline to take part in the QPE
- Birth partners
  Mother declines for birth partners to be contacted to take part in the QPE

#### Who Will Take Informed Consent?

As consent to the trial will take place in labor, the process to obtain informed consent will follow the Royal College of Obstetricians and Gynecologists guidance for intrapartum consent for research in acute situations [17]. The consent process has been shaped through the opinions of a patient and public involvement (PPI) group and the favorable use of verbal consent to emergency interventions in labor adopted by 2 preceding intrapartum studies [18,19].

In light of the findings of the internal pilot and QPE, recommendations will be made to optimize recruitment. Before labor, antenatal information will be made available to women through posters, leaflets in case notes, and a study website. Once admitted to hospital for an induction of labor or in the early stages of labor, women can be informed broadly of intrapartum studies that are taking place within the unit and that they may have the option of taking part as their labor progresses. Through these different strategies, the aim is that most women will be aware of the trial and the potential to be approached to take part in intrapartum research.

In labor, in the presence of malposition of the fetal head during the second stage, a rotational vaginal birth can be jointly agreed upon by both the woman and her clinicians. Consent to a rotational vaginal birth will then be taken, as it would be in standard clinical practice. The recruiting clinician will have an informed discussion with the woman about the option to take part in ROTATE, supplemented by the antenatal participant information sheet or concise infographic. Verbal consent to ROTATE can then be taken by an obstetrician who is Good Clinical Practice (GCP) trained. Verbal consent must be taken in the presence of a second clinician, either a physician or midwife, who is not involved with recruitment to ROTATE. Only a clear, positive “yes” from the woman will be accepted as informed consent to ROTATE. If there is any concern, uncertainty, or refusal, the woman should not be recruited to ROTATE. Routine care should proceed following this refusal. Verbal consent to the study will be documented in the woman’s clinical notes, including confirmation by the second clinician who witnessed the consent process. Randomization will be carried out only after verbal consent has been obtained. The randomization procedure undertaken by either the 24-hour telephone service or web-based system takes approximately 3 minutes to complete and does not add time pressure for clinicians. Only obstetricians listed on the training and delegation logs—who are GCP trained, familiar with the trial, and authorized to obtain consent for rotational vaginal birth—are permitted to perform the randomization. For example, a consultant could verbally consent the woman and a registrar could complete the randomization process on their behalf.

Individuals for whom the recruiter believes informed consent cannot be appropriately obtained, such as those in distress, experiencing language barriers, under time constraints, or affected by medication that may impair judgment, should continue to receive routine care.

Postnatally, women who have given verbal consent to ROTATE in labor will be approached to seek written consent. A written postnatal information sheet will be provided. This will be done before discharge from the hospital, usually within 24 to 48 hours after the birth. Written consent will include an agreement to use data already collected and to participate in follow-up assessments.

During the internal pilot, there is the additional element of being contacted and consenting to participate in the QPE for women who agreed to participate in ROTATE, as well as their birth partners and, importantly, those women who declined to participate. Clinicians involved in the internal pilot may also be approached to participate in the QPE.

The process of consent will be refined as the trial progresses, particularly during the internal pilot, with involvement from the PPI coapplicants, lay PPI panel, and senior PPI board. The findings of the QPE will play a key role in this as well.

#### Additional Consent Provisions for Collection and Use of Participant Data and Biological Specimens

Optional consent to longer-term follow-up as part of the RCT will also be taken from the woman. Similarly, participants have the option to consent to their ROTATE data being used to support future related research conducted by University College London. These data may be shared anonymously with other researchers subject to a data sharing agreement (DSA).

ROTATE does not involve any collection or storage of biological specimens.

### Intervention

#### Explanation for Choice of Comparators

Women will be randomized to either manual rotation or instrumental rotation in ROTATE. The choice of rotational instrument is up to the operator, as it is known that not all obstetricians are able to perform both Kielland rotational forceps and rotational vacuum. However, many obstetricians will be competent in the use of either rotational instrument [16]. For those unable to perform a rotational method independently, they will be directly supervised by an obstetrician who is competent in that rotational method and is GCP trained.

#### Intervention Description

Women with persistent malposition of the fetal head at full cervical dilatation are randomized to either manual rotation (intervention) or instrumental rotation (comparator: either rotational forceps or rotational vacuum).

#### Criteria for Discontinuing or Modifying Allocated Interventions

If a participant declines the rotational method they have been randomized to, the reason for the refusal and subsequent discussion with the obstetrician or midwife will be documented in the clinical notes.

Likewise, if an obstetrician feels that the randomized rotational method is not suitable or unsafe in that clinical context, a different rotational method can be used.

#### Strategies to Improve Adherence to Interventions

Adherence will be recorded using an adherence checklist in the day 0 case report form (CRF). This will include adherence to the intervention, modifications, and the reasons for this.

Adherence checklist includes the following:

1. Randomized rotational method
2. First rotational method used—including the reasons why and if it is different from the randomized method
3. Second rotational method used—if the first rotational method failed
4. Clinical reasons for the changes—to be recorded in detail

#### Relevant or Concomitant Care Permitted or Prohibited During the Trial

There will be no prohibitions or restrictions on concomitant care or recruitment to other research during the trial.

#### Provisions for Posttrial Care

Women and their babies will return to routine care following their participation in ROTATE (Table 2).

For women, the day 0 CRF will be completed by the obstetrician who performed or attempted the rotational vaginal birth. This will capture the coprimary outcomes of severe perineal trauma, defined as OASI, as well as the success of the first rotational method and the mode of birth, including cesarean birth at full dilatation.

Estimated blood loss following birth will be recorded up to 24 hours after birth, to capture primary PPH. Any blood transfusions, including red blood cell transfusions or the initiation of cell salvage of ≥300 mL, administered from randomization up to 48 hours after birth will be recorded. Likewise, at 48 hours after birth or before discharge, whichever is sooner, participants will be asked to complete an infant feeding questionnaire to capture breastfeeding outcomes, along with 2 maternal experience questionnaires—the Childbirth Experience Questionnaire (CEQ) and the Client Satisfaction Questionnaire-8 (ClisQ).

At 3 months after birth (+ or −7 days), women will be asked to complete the CEQ, ClisQ, and infant feeding questionnaires. In addition, outcomes of urinary and fecal incontinence will be captured with the International Consultation in Incontinence Questionnaire (ICIQ) and Fecal Incontinence Quality of Life Scale, respectively. Finally, posttraumatic stress disorder (PTSD) symptoms will be assessed using the City Birth Trauma Score.

For babies, a neonatal CRF will be completed by a research midwife before discharge. The ROTATE composite outcome of severe neonatal trauma and morbidity comprises any of the following:

- Stillbirth after study entry
- Early neonatal death (≤7 days)
- Evidence of intrapartum hypoxia (Appearance, Pulse, Grimace, Activity, and Respiration score ≤7 at 5 minutes after birth)
- The presence of neonatal encephalopathy receiving treatment with therapeutic hypothermia
- Neonatal seizures
- Meconium aspiration syndrome
- Brachial plexus injury, fractured humerus, or fractured clavicle

These outcomes reflect either intrapartum hypoxia or a technically difficult birth that causes physical injury to the baby. These composite outcomes are to be a safety signal, as suggested by the PPI group and perinatal experts from within the study team. All of these are considered rare neonatal events, which is why a composite outcome is used.

**Table 2:** Schedule of assessments.

Assessment			Screening		Day 0		48 hours (after maternal discharge)		Neonatal discharge		3 months + or −7 days
Eligibility check			✓		✓						
Valid informed consent^a^					✓		✓				
Randomization					✓						
**Coprimary outcomes**											
	OASI^b^			✓							
	Cesarean birth			✓							
Neonatal composite and component									✓		
Assessment of adverse events							✓		✓		✓
**Process outcomes**											
	Ultrasound use to diagnose the position before rotation	✓		✓							
	Fetal position before rotation	✓		✓							
	Fetal position at birth			✓							
	Vacuum cup position			✓							
	Forceps marks			✓							
	Adherence to the key steps of manual rotation			✓							
	Adherence to the standardized assessment of the primary outcome			✓							
**Maternal**											
	Vaginal birth after successful rotation with the first method			✓							
	Change from rotational vacuum to rotational forceps			✓							
	Severe second-degree or cervical tears			✓							
	Breastfeeding (UK Infant Feeding Survey)					✓				✓	
	Estimated blood loss					✓					
	Red blood cell transfusion or cell salvage					✓					
	Urinary incontinence (ICIQ^c^)									✓	
	Fecal Incontinence Quality of Life Scale									✓	
**Maternal experience**											
	CEQ^d^					✓				✓	
	CliSQ^e^					✓				✓	
**Maternal PTSD^f^** **symptoms**											
	City Birth Trauma Scale									✓	

^a^Valid informed consent was sought verbally on day 0.

^b^OASI: obstetric anal sphincter injury.

^c^ICIQ: International Consultation in Incontinence Questionnaire.

^d^CEQ: Childbirth Experience Questionnaire.

^e^CliSQ: Client Satisfaction Questionnaire-8.

^f^PTSD: posttraumatic stress disorder.

### Outcome

#### Primary Outcomes

The primary outcomes were OASI (coprimary superiority) or cesarean birth (coprimary noninferiority).

#### Secondary Outcomes

The secondary outcomes include successful vaginal birth with first rotational method used; neonatal morbidity, as defined earlier; maternal childbirth experience; urinary and fecal incontinence; breastfeeding; and PTSD symptoms.

#### Qualitative Process Evaluation

To explore the acceptability and feasibility of trial processes for women, their birth partners, and health care professionals will be invited to participate in the embedded QPE during the pilot phase.

### Participant Timeline

**Figure 1:**
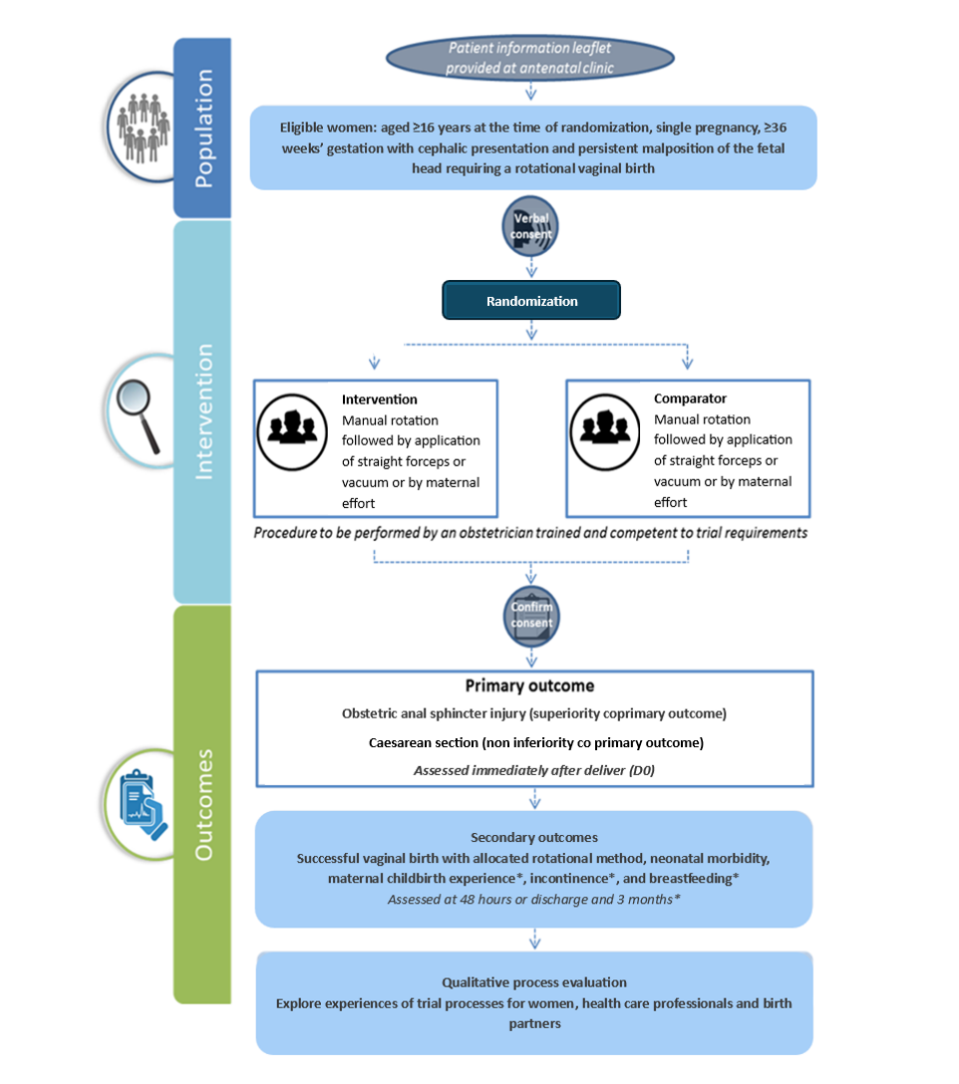
Trial Schema.

The study flowchart is presented in Figure 1.

### Sample Size

#### Main Trial

##### Superiority (OASI)

Following a meta-analysis of 5 recent studies, a pooled estimate of OASI in the control group was calculated to be 5% [4,20-23]. A recent national audit of rotational methods, Rotational Delivery at Full Dilatation, found OASI rates to be 6.6%, 4.6%, and 4.3% for Kielland rotational forceps, rotational vacuum, and manual rotation, respectively [24]. In light of this, we have estimated an incidence of OASI of 6% for the purposes of ROTATE. A 2% reduction rate of OASI was considered to be clinically significant following a consensus of experts. To detect a fall in OASI from 6% to 4% with 90% power, a 5% two-sided significance level, and using the standard method of difference between proportions based on a superiority hypothesis, the trial requires 4988 participants. We anticipate the dropout rate to be low, approximately 4%. A total sample size of 5200 participants is targeted, with 2600 allocated to each randomized arm.

##### Noninferiority (Cesarean Birth)

From the same meta-analysis, the pooled incidence for cesarean birth at full dilatation from observational data was 9%. For the control group, we estimated a conservative estimate of 12% for cesarean birth at full dilatation. Table 3 provides sample size requirements based on a 12% or 15% incidence of cesarean birth at full dilatation, calculated for 90% power and a 1-sided α of 2.5%.

If the control group rate is 15%, the planned study of 5200 participants has >90% power to detect a noninferiority margin of 3.5%. An increase of 3.5% from 15% to 18.5% represents a relative risk increase of cesarean birth at full dilatation of 23%.

**Table 3:** Sample size requirements based on 12% and 15% control group (forceps or vacuum) cesarean birth at full dilatation rates and noninferiority margins.

Control rate (%)	Noninferiority margin (%)	Total sample size (number of recruits)
12	2.9	5278
12	3	4932
12	3.5	3624
12	4	2774
15	3	5956
15	3.2	5234
15	3.5	4376
15	4	3350

#### Qualitative Process Evaluation

We plan to undertake semistructured one-to-one interviews across the sites involved in the internal pilot and will attempt to purposively recruit participants from the following groups (number of interviews per group provided in brackets):

- Women who decline to participate (approximately 5-7)
- Women randomized to the instrumental rotation group (approximately 10-14)
- Women randomized to the manual rotation group (approximately 10-14)
- Birth partners (approximately 5-7)
- Health care professionals involved in recruitment and randomization (approximately 12-15)
- Midwives involved in the care of women who are approached (approximately 8-10)

Numbers will remain flexible to ensure that the overall sample and associated data have sufficient information power to develop new knowledge in relation to the research objectives [25].

### Recruitment

#### Main Trial

Fetal malposition at full cervical dilatation is estimated to affect approximately 5% of women [2]. If a hospital has a delivery rate of 5000 births per year, this can be approximated to 417 births per month. Of these 417 monthly births, 20 would be anticipated to be affected by fetal malposition at full cervical dilatation. A total of 12 sites will be opened in the first 9 months, with 40 sites opened in total by the end of the trial. Six recruits per month per site will reach a cumulative total of 5200 recruits within 3 years of opening.

#### Qualitative Process Evaluation

##### Women

Following an approach to ROTATE, both women who have consented and declined recruitment to the trial will be asked to consider participation in the QPE. Once verbal consent to the QPE is taken by the recruiting clinician, women will be asked to complete their details on a consent to contact form, which will be subsequently shared with the QPE research team.

Site-specific screening logs and clinical notes of all women approached about ROTATE will be reviewed by research teams at local sites. If the QPE has not been documented to have been discussed, or women have asked to discuss it later, the research midwives will send a letter within 4 weeks to invite them to consider participating.

##### Birth Partners

Birth partners will be approached after a woman has agreed to this on the consent to contact form, using the same mechanisms as mentioned earlier.

##### Health Care Professionals

Both the clinicians who recruit and randomize participants as well as the midwives caring for women who are approached for ROTATE will be contacted directly by the QPE research team. They will be identified through the delegation logs, snowballing within sites, collaborator events, and established clinical networks.

### Assignment of Interventions: Allocation

#### Sequence Generation

Women are randomized to either manual rotation or instrumental rotation at the level of the individual at a 1:1 ratio. A minimization procedure using a computer-based algorithm will be used to avoid chance imbalances in important stratification variables. Strata used in the minimization will be center and fetal position (occiput transverse or occiput posterior).

#### Concealment Mechanism

There will be no concealment mechanism in ROTATE, as it is an open-label RCT.

#### Implementation

Once eligibility has been confirmed and verbal consent has been obtained as previously outlined, a physician is GCP trained and on the delegation log can undertake the randomization process. Randomization will be carried out in 1 of 2 ways. Either, a 24-hour automated telephone service can be used. Alternatively, web-based randomization through a website can be performed. Either method of randomization will ask 5 questions confirming eligibility and the center identification number. The allocated rotational method will then be generated and given to the clinician, either spoken through the telephone service or displayed on the website.

### Assignment of Interventions: Blinding

#### Who Will Be Blinded?

ROTATE is an unblinded RCT, so no participants or clinicians will be blinded.

#### Procedure for Unblinding

As there will be no blinding, unblinding will not occur.

### Data Collection and Management

#### Plans for Assessment and Collection of Outcomes

The day 0 CRF will capture the coprimary outcomes of OASI, diagnosed on clinical vaginal or rectal examination immediately after birth, and cesarean birth.

Other information captured on this form includes the following:

- The level of the obstetrician performing or supervising the rotation
- The obstetrician’s preferred rotational method
- How fetal malposition was identified—through vaginal examination alone or with intrapartum ultrasound
- The first rotational method attempted, and if it differed from the randomized method, the reasons for this deviation
- If the first rotational method was successful
- Whether a second rotational method was attempted
- The mode of birth following successful or unsuccessful rotation (direct forceps, direct vacuum, maternal effort, or cesarean birth)
- The position of the fetal occiput or posterior fontanelle at birth

Within 48 hours of birth, a research midwife will complete forms documenting maternal blood loss and neonatal outcomes. Women will be asked to complete the CEQ, ClisQ-8, and infant feeding forms at 48 hours or before discharge, whichever is sooner. Finally, 3 months after birth, participants will be approached to complete the CEQ, ClisQ-8, and infant feeding forms again, as well as the ICIQ, the Fecal Incontinence Quality of Life Scale, and City Birth Trauma Scale. These forms can either be sent by post to women or completed on the web after being contacted via email.

#### Plans to Promote Participant Retention and Complete Follow-Up

Women will be supported during the recruitment process by information being shared by members of the research team and supported with antenatal information, including the infographic during labor. They will be informed that they may decline recruitment or withdraw their consent at any time, without providing a reason, and that doing so will not affect the care that they will receive.

To encourage retention, members of the local research teams will aim to obtain written consent from participants before discharge. Although this will usually be done by a research midwife, physicians will be asked to take written consent if it is unlikely that a research midwife will be able to speak to the participant before discharge, for example, a participant recruited on a Friday evening who will be returning home before Monday morning.

To complete the follow-up, women can be contacted once by the local research teams with either a letter or a telephone reminder if no response has been received within 2 weeks.

### Data Management

#### Day 0 CRF

Data will be captured on the day 0 CRF, which can be either a paper questionnaire or web-based submission using REDCap (Research Electronic Data Capture; Vanderbilt University) or using a web-based patient record, if the local unit has this facility. REDCap is a secure, dedicated web-based data capture system.

#### Follow-Up at 48 Hours and 3 Months

The 48-hour and 3-month questionnaires, including the Infant Feeding Survey, Neonatal CRF, maternal estimated blood loss, CEQ, ClisQ-8, ICIQ, Fecal Incontinence Quality of Life Scale, and City Birth Trauma Scale, will be completed using the participant’s preferred method—either letter, telephone, email, or on the web. Paper questionnaires will be sent by post to the local research team.

Data gathered through paper questionnaires will be transcribed onto the REDCap database by the local research teams. Paper copies of any forms will be retained at the site. The local principal investigators (PIs) will be responsible for ensuring that trial documents from their units are securely retained. Data, both paper and electronic, will be stored confidentially for 25 years.

### Confidentiality

#### Main Trial

ROTATE will be registered with the data protection officer at the University of Birmingham. Data will be held in accordance with the Data Protection Act (2018).

Participants’ anonymity will be maintained by the research teams. At randomization, an identification number will be generated, which will be used during data collection. Only study investigators will have access to the dataset stored on the secure database. Only if there is a regulatory or institutional requirement, such as for data entry quality checks, would there be an exception. Database passwords will not be shared.

The relevant site staff will undergo study-specific data entry training at the site initiation visit or through web-based training. This will be supported by written guidance. Any consent forms or identifiable data can be sent via email only through a secure National Health Services email provider (@nhs.net email address or equivalent).

#### Qualitative Process Evaluation

Written informed consent will be taken where possible. In cases where study-related paperwork has either not been received or completed fully, or there are concerns regarding written literacy, alternate forms of consent will be sought. These include electronic consent, where the form is completed electronically and returned as a scan or photo, and verbal consent, where the form is read aloud in full and audio recorded as a separate file at the start of the interview. With verbal consent, if a participant declines to consent to any of the statements, the interview will be terminated at that time.

Informed consent, no matter the form in which it is taken, will include agreement to participate, demographic data collection, audio-recorded discussion, and anonymized data sharing.

At the beginning of the audio recording, verbal reconfirmation of consent will be taken from participants who have completed electronic or written consent, and then, this recording will be closed. For participants who have given consent, a second audio recording will then commence for the interview. A member of the QPE research team will transcribe the consent audio file to generate a formal record of informed or declined consent. The second audio file of the interview will be sent to a third-party company for transcription. The consent audio file will be stored securely and separately from the transcript of the main interview if consent was gained. Interview participants will be able to withdraw their consent within 2 weeks of data collection without needing to provide a reason.

### Plans for Collection, Laboratory Evaluation, and Storage of Biological Specimens for Genetic or Molecular Analysis in This Trial or Future Use

No biological specimens will be collected during the ROTATE trial.

### Statistical Methods

#### Statistical Methods for Primary and Secondary Outcomes of the Main Trial

##### Overview

Participants will be randomly allocated into 2 groups: manual and instrumental rotation. All participants’ data will be analyzed based on the group they have been allocated to, regardless of any protocol deviations or adherence to the randomization.

Appropriate summary statistics and differences between groups will be presented for all relevant outcomes along with 95% CIs, and *P* values from 2-sided tests will also be provided. Where possible, intervention effects will be adjusted for the minimization (hospital and fetal position—occiput posterior or occiput transverse). No adjustment for multiple comparisons will be made.

##### Primary Outcome

The coprimary outcomes of OASI and cesarean birth at full dilatation will be analyzed using mixed-effects log-binomial models. This will generate relative risks alongside risk differences with 95% CIs, adjusting for fetal position as a fixed effect and hospital as a random effect. A Poisson regression model with robust SEs will be used to estimate the same parameters if the log-binomial model fails to converge. Should a Poisson regression model also fail, unadjusted estimates will be produced from the log-binomial model.

As a joint effect of both outcomes is not being assessed, type II error adjustments have not been applied. Interpretation of the results will be based upon the treatment effect and 95% CIs for each of the coprimary outcomes.

##### Secondary Outcomes

The same analysis will be performed for all binary secondary outcomes (such as successful vaginal birth and breastfeeding intention after birth) as for the primary outcome, as well as any safety output, including serious adverse events (SAEs). For continuous outcomes (eg, estimated blood loss), a mixed-effects linear regression model will be used to generate differences between group means and CIs, adjusting for the same minimization parameters as the primary outcome.

#### Statistical Methods for Primary and Secondary Outcomes of the QPE

To analyze the qualitative data, the framework approach will be used to facilitate a systematic and flexible approach. This aims to identify thematic patterns and commonalities while accounting for individual variation in experience within the interpretive account. Meanings will be generated and constructed through situational, interpretative engagement with the data. This will produce dynamic feedback that can be shared with the Trial Management Group (TMG) during the pilot phase. This is aimed to improve and streamline processes of the trial and ensure that lessons learned continuously enhance the pilot.

### Interim Analyses

#### Main Trial

During the trial, interim analyses of both efficacy and safety will be presented to the independent Data Monitoring Committee (DMC). Analysis of the coprimary and significant secondary outcomes, as well as a full assessment of safety from SAEs, will likely be included in at least annual reviews. On the basis of this information, criteria for stopping or modifying the trial will be ratified by the DMC.

#### Qualitative Process Evaluation

Data from the QPE in the pilot will be shared with the Trial Steering Committee (TSC) to support their decision-making about progression to the full trial.

### Methods for Additional Analyses, Such as Subgroup Analyses)

Subgroup analyses will be performed on the coprimary outcomes, including division of instrumental rotation into Kielland rotational forceps and rotational vacuum. However, this will be limited to the same variables from minimization. By including an intervention group by subgroup interaction parameter in the regression model, the effects of the subgroups will be analyzed. This will be presented alongside the effect estimate and 95% CI within the subgroups. It should be noted that the results of the subgroup analyses should be interpreted with caution. These results will only be used to generate, rather than confirm, hypotheses.

### Methods in Analysis to Handle Protocol Nonadherence and Any Statistical Methods to Handle Missing Data

As much as possible, attempts will be made for full follow-up data to be collected from all ROTATE participants. In light of this, it is expected that there will be minimal missing data. Participants with missing primary outcome data will not be included in the primary analysis. However, this would present a risk of bias. To assess the possible impact of this risk of bias, sensitivity analyses will be carried out.

The 2 randomized groups will be manual rotation and instrumental rotation. The randomized allocation is the first rotational method to be attempted. In cases where the randomized rotational method fails, some crossover is expected, such as switching to instrumental rotation after failed manual rotation, or vice versa. However, this is expected to be low based on a crossover rate of 0.6% in 2 recent studies [4,20]. Using appropriate sensitivity analyses, including per protocol analysis of the 2 coprimary outcomes, we will examine the robustness of the conclusions.

Adherence to the allocated rotational method will be recorded on the adherence checklist within the day 0 CRF. Videos to standardize the techniques for the 3 rotational methods will be made available to recruiting sites before the trial opens. These can be shared by the local PI [26].

### Plans to Give Access to the Full Protocol, Participant-Level Data, and Statistical Code

The protocol is available to the public. Trial-related monitoring, audits, ethical review, and regulatory inspections will be permitted by the investigator. This will include direct access to source data and documents.

Access to the anonymized trial data will be considered by the Birmingham Clinical Trials Unit (BCTU) typically within 6 months after the primary publication. Only scientifically sound proposals from appropriately qualified research groups will be considered for data sharing after review by the BCTU Data Sharing Committee in discussion with the chief investigator. In the absence of the chief investigator or where appropriate, discussions will include any of the TSC, TMG, and independent TSC. Once the release of the data is approved and before the data are released, a formal DSA may be required between the respective organizations. All data will be anonymized unless the DSA includes the transfer of identifiable patient information. Transfer of data will be through a secure and encrypted method.

Participants will be told in the information sheet that this may happen and asked to verify they understand this in the consent form. The findings of ROTATE will be published in peer-reviewed journals. Likewise, the results will be shared with participants who wish to be informed.

### Oversight and Monitoring

#### Composition of the Coordinating Center and TSC

The TSC will comprise 75% independent membership, including an independent chair (UK-based or holding a substantive UK-based appointment); an independent statistician or methodologist; ≥2 PPI members from PPI coapplicants, the lay PPI panel, and the senior PPI board; and ≥1 topic expert. The TSC will invite observers, including a representative of the sponsor and a representative from the research network, to meetings as appropriate. Attendance at TSC meetings by nonmembers will be at the discretion of the chair.

#### Composition, Role, and Reporting Structure of the DMC

The DMC will include topic and methodology experts, a statistician, and ≥1 member who is UK-based or holding a substantive UK-based appointment. The DMC will have 3 to 4 independent members, in line with the National Institute for Health and Care guidance. The DMC will meet at least annually. The DMC’s main roles will include the following:

- Being the only body with access to unblinded comparative data
- By monitoring these data, they can make recommendations to the TSC with any ethical or safety reasons that should stop the trial from continuing
- To consider whether any interim analysis is necessary to advise the TSC on the release of data or information
- If asked by the TSC, project sponsor, or project funder, the DMC could consider data coming to light from other related studies. If there are serious concerns regarding the viability of ROTATE, the DMC chair may be asked by the project funder to produce an interim or futility analysis. The same may be asked if the research team is requesting significant extensions.

### Adverse Event Reporting and Harms

#### Adverse Events

Adverse event (AE) reporting will be in accordance with the UK Policy Framework for Health and Social Care Research (2017), GCP, and the requirements of the Health Research Authority. The local research teams will document AEs in participants’ notes. This will include the severity and seriousness of harm, as well as a relationship to any interventions. The reporting period for AEs will be 3 months and 7 days after the birth. Most AEs will be captured on the CRFs.

#### Serious AEs

When an SAE occurs, the PI or any authorized person listed on the delegation log at the site should first report the SAE to their Trust according to local procedures. Next, the PI or delegates should complete an SAE form, which is signed and dated by the PI. A copy of the SAE form along with any relevant anonymized patient data should be sent by the PI to inform BCTU of the SAE according to the following criteria:

1. Record safety reporting-exempt SAEs in the medical notes (including preplanned hospital admission and hospital admission of the mother for <24 hours).
2. Report SAEs to the trial office in a nonexpedited manner (within 4 weeks of the local research team becoming aware of the event; including unplanned hospital admission of the baby within the follow-up period and unplanned admission of the mother for >24 hours during the follow-up period).
3. Report SAEs to the trial office in an expedited manner (within 24 hours of the local research team becoming aware of the event).

This includes any other SAE that has not been listed earlier.

The PI or delegate who completes the SAE form should indicate the seriousness and causality of the event (Table 4).

Once the SAE has been received by BCTU, the chief investigator or delegate will independently review the causality. An SAE deemed by the chief investigator, PI, or delegate to have a reasonable causal relationship with an intervention will be viewed as a related SAE. Should the chief investigator and PI disagree on the causality assessment, both opinions will be documented. Should the event require further reporting, the relevant opinion will be included with the report.

The chief investigator or delegate will assess the expectedness of an SAE (Textbox 3).

BCTU will report any event deemed to be unexpected and related SAEs to the Research Ethics Committee (REC) and sponsor within 15 days of BCTU being notified. However, if any significant safety concerns are suspected or found during the course of ROTATE, the main REC and the University of Birmingham Research Governance Team will be notified immediately for their advice. Should an urgent safety measure be taken, BCTU will immediately (within 3 days) give written notice to the REC of the actions taken and the reasons for this.

After an SAE has been reported, the participant should be followed up until the event has resolved or stabilized. Further information should be sent to BCTU as required.

**Table 4:** Serious adverse events relatedness categories.

Category	Definition	Causality
Definitely	Clear evidence suggesting a causal relationship, and other possible contributing factors can be ruled out.	Related
Probably	Evidence suggesting a causal relationship, and the influence of other factors is unlikely.	Related
Possibly	Some evidence suggesting a causal relationship. However, the influence of other factors may have contributed to the event.	Related
Unlikely	Little evidence suggesting a causal relationship. There is another reasonable explanation for the event.	Unrelated
Unrelated	No evidence of any causal relationship.	Unrelated

**Serious adverse events’ expectedness categories.**

**Category and definition**

- Expected: adverse event consistent with known information from manual or instrumental rotation
- Unexpected: adverse event that is inconsistent with known information from manual or instrumental rotation

### Frequency and Plans for Auditing Trial Conduct

Trial-related monitoring and audit, ethical review, and inspection will be permitted. No routine monitoring visits are planned. However, on-site monitoring will be undertaken if there is a cause for concern for BCTU, such as consistent poor data returns or large volumes of protocol deviations.

### Plans for Communicating Important Protocol Amendments to Relevant Parties, Such as Trial Participants and Ethical Committees

All amendments will be documented in the protocol under the “Protocol Amendments” section. Decisions to amend the protocol and any trial documents will be initiated by the TMG. University College London, as the sponsor, will deem whether an amendment is either substantial or nonsubstantial.

To make substantial changes, a submission will be required to be sent to the REC and Health Research Authority for their approval. Once received, Research and Development departments at local sites will be informed of the proposed amendment and their approval requested. If no response has been received from a site after 35 days, it will be assumed that they do not object to the amendment, and it will be implemented.

### Ethical Considerations

Ethics approval to conduct the trial has been obtained from London—Surrey REC (REC reference 22/LO/0157; Integrated Research Application System, IRAS 301912) on May 19, 2022. Verbal consent will be taken at the point of recruitment once eligibility has been confirmed and in the presence of a clinician (physician or midwife) who is unaffiliated with the trial. Written consent will be obtained within 48 hours of birth. Separate informed consent will be sought for participation in the qualitative process evaluation. The privacy and confidentiality of recruits’ data and identity has been maintained. No compensation was awarded to recruits.

### Dissemination Plans

The findings of ROTATE will be disseminated through several channels to reach a wide audience. The publication policy will be overseen by the TSC.

#### Public Engagement

A dedicated website will be created to share the progress and findings of ROTATE. The PPI partners will distribute the results of the study to parents in their networks. Finally, the study team will disseminate short videos, graphics, and vignettes summarizing the trial findings on their social media platforms (X [X Corp], Facebook [Meta Inc], Instagram [Meta Inc], and Mumsnet).

#### Professional Stakeholder Engagement

By working with the National Institute for Health and Care research networks, Academic Health Science Networks, and Applied Research Collaborations, the findings will be available as evidence to be critically appraised and subsequently used to update guidance for rotational vaginal birth from the Royal College of Obstetricians and Gynecologists and National Institute for Health and Care Excellence.

#### Academic Stakeholders and Outputs

It is anticipated that the results will be published in peer-reviewed journals with a high-impact factor. We aim to present at national and international conferences to share the learnings within the academic community.

#### Pathway to Practice Improvement

The chief investigator is the convenor of RCOG Assisted Birth Simulation Training, a national mandatory course of assisted vaginal birth for UK trainees. Key findings from the trial will inform future iterations of the course, including emphasizing which rotational method has been found to be safest and most efficacious, and recommendations for consent in labor. Members of the study team also organize a national course on rotational vaginal birth, Advanced Rotational Techniques and Safe Caesarean Birth at Advanced/Full Dilatation Training, which is aimed at senior trainees and consultants [27]. Likewise, the findings of ROTATE will be highlighted in the same way.

#### Authorship

There are no plans for the intended use of professional writers. Authorship will be justified to and agreed upon by the TMG. The TSC will resolve any difficulties or disagreements. If any individual members of the group are not satisfied with a decision, they can appeal to the TMG for reconciliation. If this cannot be resolved, it will be referred to the TSC.

## Results

Recruitment started on September 9, 2022, and ended on November 5, 2024. A total of 321 participants were recruited. After review with the funder and due to not meeting the anticipated recruitment targets, ROTATE was closed. The trial closed on February 9, 2025. Data analysis of primary and secondary outcomes, as well as novel findings, is ongoing at the time of submission.

The protocol used for the submission is version 5.0, dated December 15, 2023.

## Discussion

### Principal Findings

Fetal malposition in the second stage of labor increases the likelihood of obstetric intervention, including rotational vaginal birth and cesarean birth at full dilatation. Rotational vaginal birth risks OASI and neonatal trauma [18]. However, cesarean birth at full dilatation carries greater chances of some adverse maternal and neonatal outcomes compared with both cesarean birth before or at an earlier stage of labor as well as assisted vaginal birth [8,9,12].

ROTATE is a pragmatic, multicenter, open-label, 2-arm parallel group RCT of manual rotation versus instrumental rotation of the fetal head in persistent malposition at full cervical dilatation. We hypothesize that manual rotation will have a lower rate of OASI without substantially increasing the chance of cesarean birth at full dilatation when compared with instrumental rotation. The choice of instrument, Kielland rotational forceps or rotational vacuum, is up to the operator. We aim to recruit 5200 participants over a 3-year period at approximately 40 consultant-led maternity units across the United Kingdom. The coprimary outcomes, OASI and cesarean birth at full dilatation, will be recorded immediately after birth. Secondary outcomes of maternal blood loss in the first 24 hours, neonatal trauma, breastfeeding, and birth experience will be collected at 48 hours after birth or before discharge. Further secondary outcomes of breastfeeding, birth experience, PTSD, and urinary and fecal incontinence will be collected at 3 months after birth.

Improved evidence to support rotational vaginal birth is hoped to improve obstetric practice worldwide. Likewise, it aims to directly address harrowing reports of birth trauma, OASI and its long-term detrimental impact on quality of life, and neonatal trauma following assisted vaginal birth [28].

Strengths of the study include its multicenter RCT design. No RCT of rotational methods has ever been completed, leaving a gap in this level of robust evidence. It is also unlikely that a multicenter RCT of rotational methods could be repeated anywhere in the world. Limitations include selection bias, as both the sites participating in ROTATE and the clinicians recruiting are likely to have a greater interest in and potentially more skill with rotational vaginal birth than other obstetricians. The results may be less applicable to those with limited experience and confidence in rotational vaginal birth. As the trial is unblinded, it could lead to biased assessment of subjective outcomes, including OASI diagnosis, considering the same clinician may perform and assess the procedure. Although the instrumental rotation arm includes both Kielland rotational forceps and rotational vacuum, which can introduce heterogeneity, secondary analyses will be able to determine additional details regarding the instrumental rotational used and subsequent outcomes.

### Conclusions

A multicenter RCT of rotational methods has been conducted in the United Kingdom with the primary outcome of determining whether manual rotation reduces the risk of severe perineal trauma without increasing the risk of cesarean birth, compared with instrumental rotation. Although the trial was closed early due to challenges in meeting recruitment targets, the results are still highly anticipated, with potential implications on the management of fetal malposition at full cervical dilatation and rotational vaginal birth in the United Kingdom and globally. Lessons learned from conducting this intrapartum RCT, including insights from the embedded QPE, aim to guide much-needed future intrapartum studies.

## Abbreviations

AE: adverse event
BCTU: Birmingham Clinical Trials Unit
CEQ: Childbirth Experience Questionnaire
ClisQ: Client Satisfaction Questionnaire-8
CRF: case report form
DMC: Data Monitoring Committee
DSA: data sharing agreement
GCP: Good Clinical Practice
ICIQ: International Consultation in Incontinence Questionnaire
OASI: obstetric anal sphincter injury
PI: principal investigator
PPH: postpartum hemorrhage
PPI: patient and public involvement
PTSD: posttraumatic stress disorder
QPE: qualitative process evaluation
RCT: randomized controlled trial
REC: Research Ethics Committee
REDCap: Research Electronic Data Capture
SAE: serious adverse event
TMG: Trial Management Group
TSC: Trial Steering Committee

## Data Availability

The datasets generated or analyzed during this study are available from the corresponding author on reasonable request.
